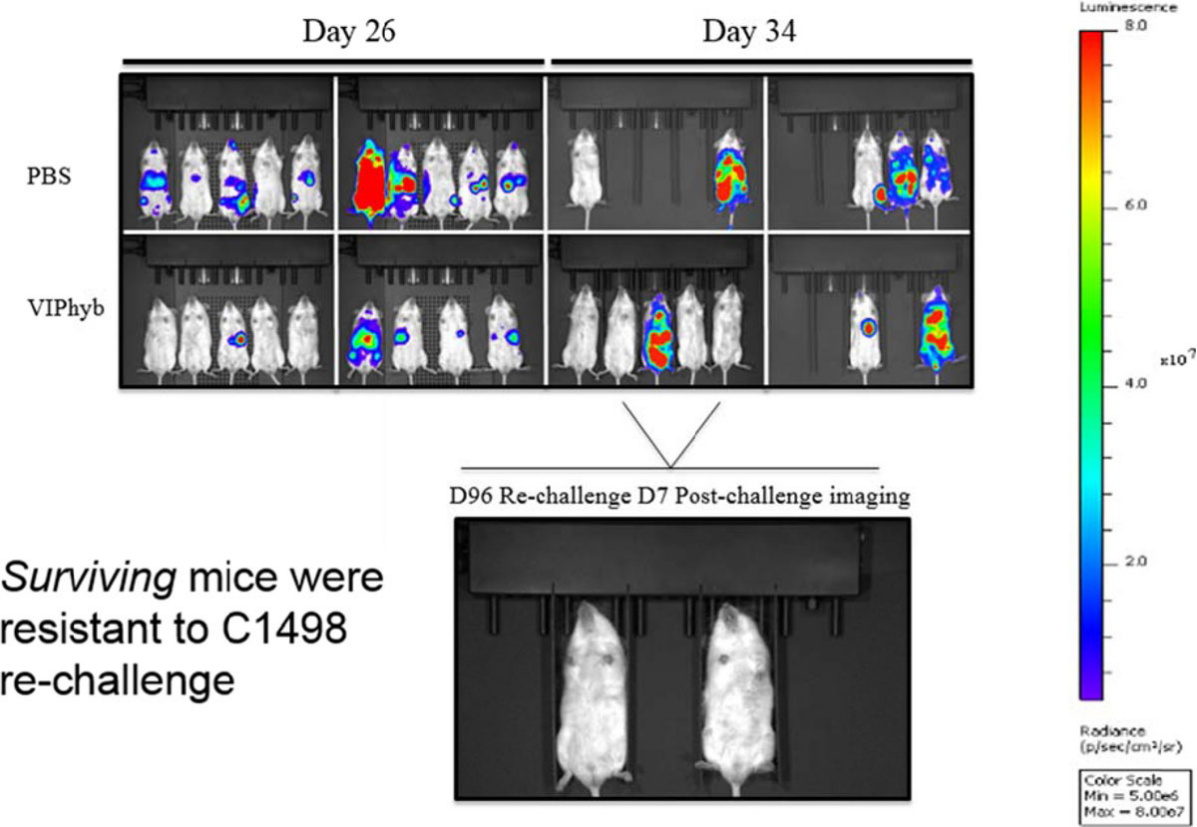# Administration of a vasoactive intestinal peptide antagonist significantly enhances the autologous anti-leukemia T cell response in a murine model of AML

**DOI:** 10.1186/2051-1426-3-S2-P238

**Published:** 2015-11-04

**Authors:** Christopher Petersen, Jian-Ming Li, Edmund Waller

**Affiliations:** 1 Emory University, Atlanta, GA, USA

## 

Products secreted from nerve terminals can have profound effects on the phenotype and functioning of T cells. One such product, vasoactive intestinal peptide (VIP) is a small neuroendocrine peptide hormone with potent anti-inflammatory activities. Binding to either of its type B G protein coupled receptors VPAC1 and VPAC2 initiates a signaling cascade that ultimately inhibits the secretion of pro-inflammatory cytokines and reduces proliferative capacity via inhibition of NF-κB[1]. The anti-inflammatory effects of VIP signaling in immune cells have demonstrated to be inhibitable with the use of one of several small molecule antagonists. VIPhyb is a hybrid peptide antagonist consisting of the VIP peptide with a six amino acid substitution at the N-terminus. We demonstrate here that subcutaneous administration of small doses of VIPhyb enhanced the autologous T cell response in mice bearing C1498 acute myeloid leukemia. Secretion of both IFN-γ and TNF-α was significantly increased in VIPhyb-treated mice. Additionally, the frequency of PD-1-expressing CD4 and CD8 T cells was significantly lower in mice treated with the VIP antagonist. This was determined to be of relevance in this model as PD-L1 was found to be inducible on C1498 cells. This expression was not affected by VIPhyb treatment. Each of these effects contributed to a reduction in tumor burden in VIPhyb-treated mice as evidenced by bioluminescent imaging. No direct cytotoxic effect of VIPhyb on the tumor cells themselves was observed despite the expression of VPAC2. Overall survival of mice that received 1, 3, or 7 doses was significantly higher than vehicle-treated controls as a result of enhanced immunological function. Importantly, VIPhyb-treated survivors were found to be resistant to further tumor development following re-challenge with C1498 cells. This observation highlights a contribution of VIP signaling blockade in the development of anti-leukemia T cell memory. The data heretofore described highlight the contribution of VIP to the inhibition of anti-leukemia T cell responses and thus present rationale for considering this pathway in the design of therapeutic strategies for AML. VIP signaling blockade is thus a promising candidate for the enhancement of immunotherapies that seek to strengthen the anti-leukemia T cell response.

**Figure 1:**
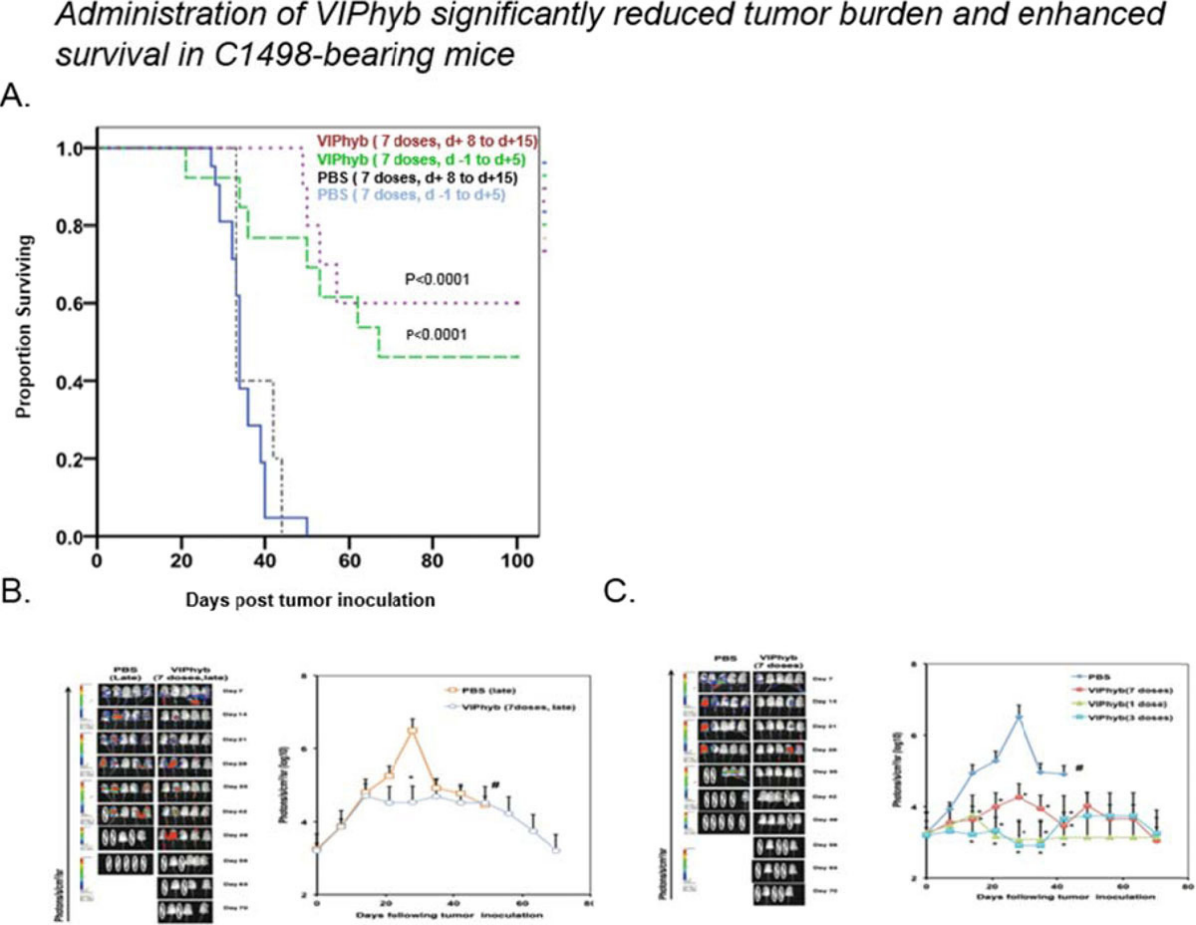


**Figure 2:**
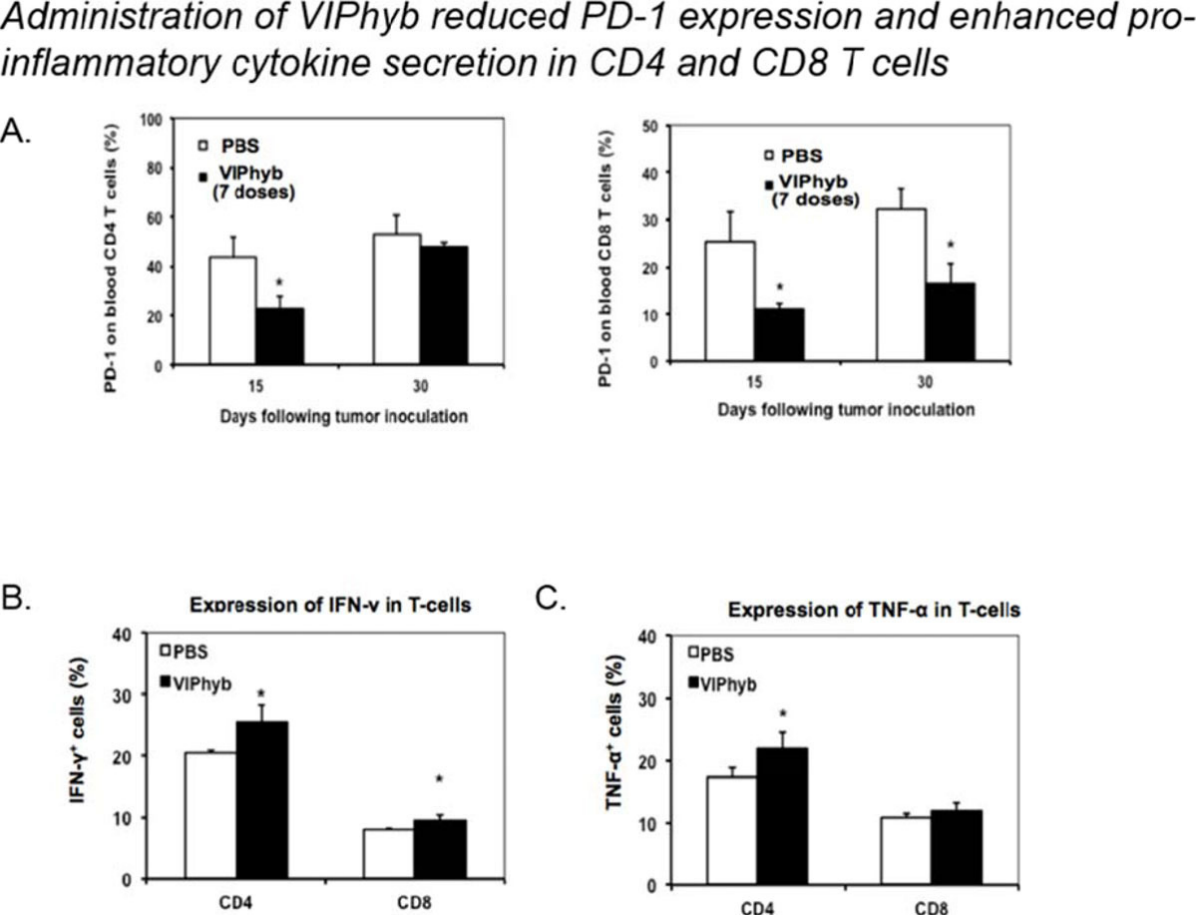


**Figure 3:**
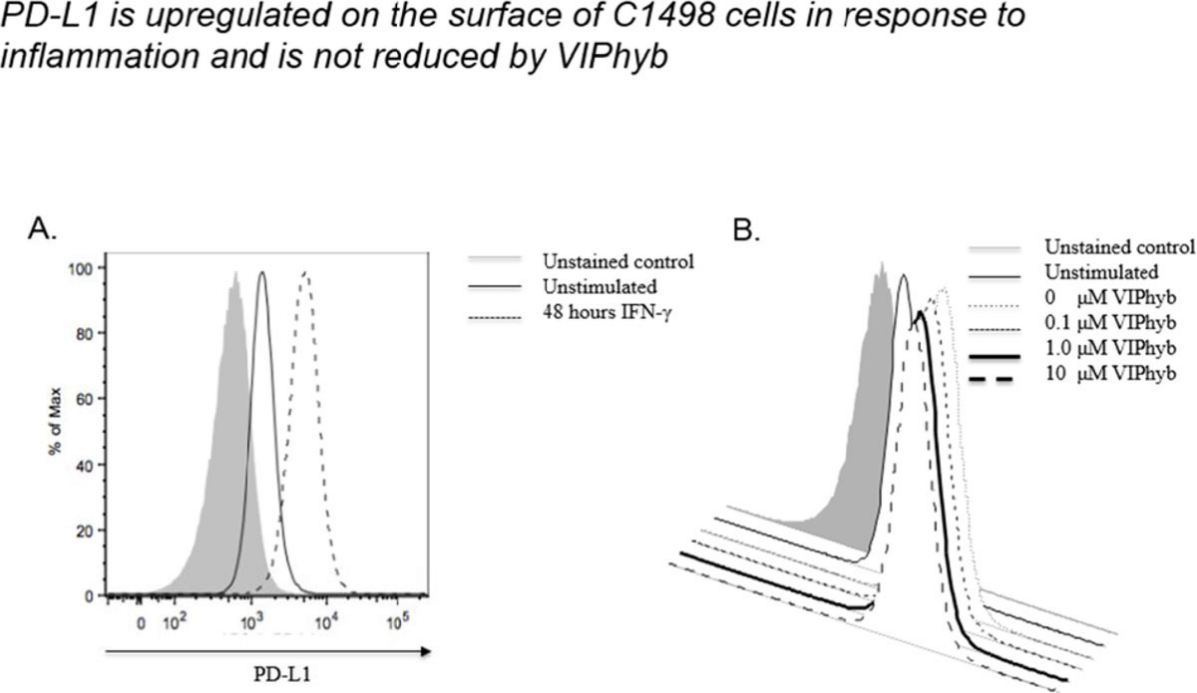


**Figure 4:**
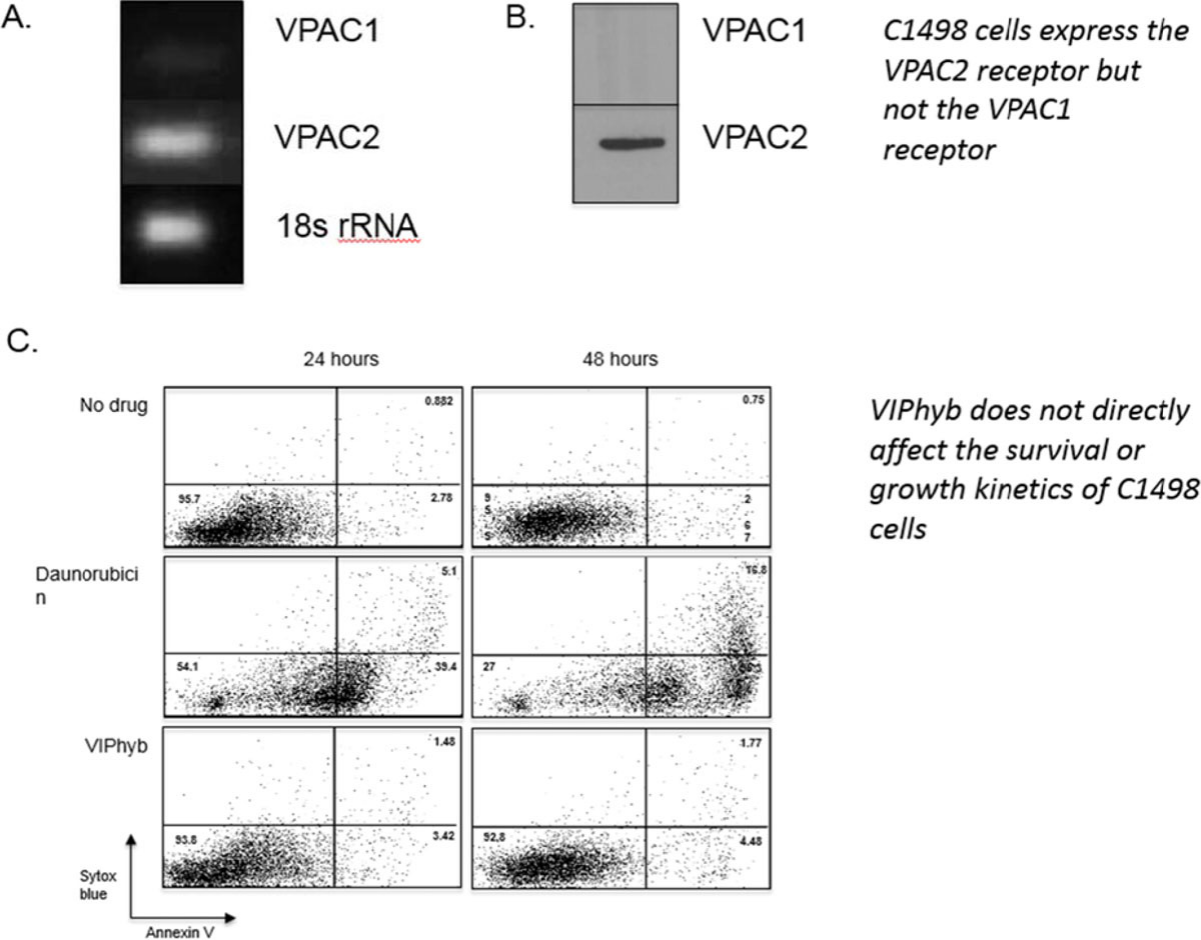


**Figure 5:**